# Exploring Constraints and Sport Experiences: A Case Study of Adult Workers in Singapore

**DOI:** 10.3390/bs14090848

**Published:** 2024-09-20

**Authors:** Heetae Cho, Ghazanfar Ali Abbasi, Hyoung-Kil Kang, Ye Hoon Lee

**Affiliations:** 1 Department of Sport Science, Sungkyunkwan University, Suwon 16419, Republic of Korea; htcho@g.skku.edu; 2 Department of Physical Education and Sport Science, Nanyang Technological University, Singapore 637616, Singapore; 3 Management and Marketing Department, KFUPM Business School, King Fahd University of Petroleum and Minerals, Dharan 31261, Saudi Arabia; 4 Department of Physical Education, Kyungnam University, Changwon 51767, Republic of Korea; 5 Division of Global Sport Industry, Hankuk University of Foreign Studies, Yongin 17035, Republic of Korea

**Keywords:** constraint, sport experience, nostalgia, satisfaction, sport involvement

## Abstract

Although rapid economic growth can produce various positive outcomes, the fast-paced society that inevitably accompanies it often results in longer working hours and higher stress levels, leading to reduced participation in sport activities among employees. To better understand this phenomenon, we aimed to explore the constraints and experiences of adult workers. We collected data from adult workers in Singapore who desired to participate in sport activities but were unable to do so due to various constraints. A total of 10 individuals were purposefully selected for interviews. This study identified four key themes: (a) factors causing the reduction of sport participation opportunities, (b) changes in sport activities and satisfaction, (c) nostalgic feelings associated with sport activities, and (d) the role of nostalgia in enhancing sport involvement. The findings of this study suggest the possibility of adapting the conceptual model of nostalgia to sport activities, as well as identifying four factors that hinder employees from engaging in sport activities.

## 1. Introduction

Sport is a significant aspect of life [1,2] that individuals engage in via personal choice due to the inherently exciting nature of these activities (i.e., intrinsic motivation) and their potential for personal development [3]. Researchers have noted that sport participation benefits an individual’s health [4,5]. For example, Passmore [6] found that sport activities could improve mental health and social inclusiveness, encourage self-expression, and foster an individual’s potential. Additionally, sport activities help prevent stress and aid in coping with it effectively [7,8,9], making them beneficial for psychological well-being [10,11] and therapeutic for psychological therapy [5].

Active sport participation also has a positive effect on participants’ physical health [12,13]. Previous research has shown that exercise can reduce the mortality rate by up to 50% for individuals who shift from a sedentary lifestyle to moderate activity [14]. Thus, sport activities that include physical activity enhance physical fitness and reduce the risk of mortality [15,16]. In summary, active sport participation can result in multiple benefits for individuals, including physical and psychological health, overall well-being, and meaningful activities with cumulative effects [17,18].

In the past, leisure was often viewed negatively, as it was thought to be in conflict with work [19]. This perspective, rooted in an industrialized worldview, has persisted in our society, particularly in Singapore, where work is often given greater priority [20,21]. Although this perception has evolved over time, it remains a prominent feature of Singaporean culture. Comparing Singapore’s work culture with that of the United Kingdom, which is often seen as representative of Western culture, can shed light on this issue. According to data from the UK Office for National Statistics, the average working hours per week in 2023 were 36.2 h, while in Singapore the average was 44.2 h [22,23]. Additionally, the GDP per capita ratios of Singapore and the UK were SGD 52,962 and SGD 40,367, respectively [24]. Based on these statistics, it can be inferred that Singapore’s higher GDP was the result of a proportionally larger workforce with longer working hours, resulting in less leisure time for individuals working full-time [25]. Sport Singapore reported that 51% of participants lacked sufficient time to engage in sporting activities due to work, while others cited family or work commitments or a lack of interest [26]. This suggests that individuals in Singapore, a highly work-oriented nation, may have limited opportunities to engage in sport activities and may be unable to fully enjoy them. Although sport can offer numerous benefits to individuals [4], many face constraints that prevent them from participating fully, such as cost, time constraints, the availability and quality of sport facilities, social and geographical isolation and personal skills and abilities [27,28,29,30].

Researchers have found that long working hours and high stress levels are associated with an increased risk of cardiovascular disease, morbidity, disability [31,32,33] and common mental disorders [34,35]. The intensive working culture in Singapore has reduced workers’ leisure time, leading to constraints that may have negative consequences, such as a decline in physical and mental health [36]. The primary research gap this study addresses is the lack of focused investigation into the specific constraints faced by adult workers in Singapore regarding their sport participation. While existing research has examined constraints in various countries, including the impact of work culture on leisure activities [37,38,39,40], there is limited empirical evidence on how the unique work environment in Singapore—a high-pressure, high-hour culture—specifically affects sport participation. Thus, this study aims to fill this gap by providing an in-depth exploration of how working adults in Singapore navigate and experience constraints that lead to reduced physical activity. In addition, Singapore’s high-pressure work culture significantly impacts the work-life balance of its residents. This work-life balance refers to the equilibrium between professional commitments and personal time, which enables participation in leisure activities, such as sport [41,42]. The tension between work and leisure is particularly acute in Singapore, where the demands of employment frequently leave little room for personal pursuits [43]. Furthermore, Godbey et al. [44] noted that individuals in different social, cultural, and historical contexts experience different constraints. Therefore, this study identifies the causes of reduced sport activities among participants in Singapore and explore their psychological responses to such constraints, such as satisfaction, nostalgia, and sport involvement. In particular, recent research has highlighted nostalgia as a key emotional factor influencing behavioral outcomes [45]. For adult workers facing various physical and psychological obstacles to sport participation, nostalgia may serve as a unique motivational tool. By evoking memories of previous engagement in sport or leisure activities, nostalgia can rekindle a desire to re-experience joy and satisfaction of physical movement [45].

This study explores how nostalgia can help buffer and overcome such barriers, positioning it as a central motivational factor for re-engagement in sport among adult workers in Singapore. To further deepen the investigation into the phenomenon of reduced sport participation among adult workers in Singapore, the following research questions were developed: (1) What are the specific constraints faced by adult workers in Singapore regarding sport participation? (2) How does nostalgia function as a motivational tool for overcoming barriers to sport participation among Singaporean workers? These research questions allow for a comprehensive exploration of the phenomena and provide important insights into the complex interplay between work-related pressures, cultural influences, and sport participation. The findings illuminate the distinctive challenges encountered by individuals in the Singaporean context, which may diverge from those experienced in other cultural milieus. By elucidating the specific constraints and their psychological ramifications, such as satisfaction and nostalgia, this study offers invaluable perspectives on the relationship between work and leisure in a highly work-centric society. This understanding is crucial for developing targeted interventions to foster enhanced sport participation and overall well-being among workers facing similar high-pressure environments.

## 2. Literature Review

### 2.1. Constraints

Constraint refers to the factor that limits an individual’s ability to participate in sport activities [46,47]. This concept has been of interest to scholars for several decades. Crawford et al. [48] proposed the earliest theory of constraints, suggesting that constraints could be categorized into three types: intrapersonal, interpersonal and structural. Intrapersonal constraints relate to factors within an individual, such as lack of skills, interests or motivation [47,49]. Interpersonal constraints relate to factors in a person’s social environment, such as lack of social support or conflicts with others [46,50]. Structural constraints refer to factors outside of an individual’s control, such as lack of time, money or access to sport facilities [48]. Time and money are commonly cited structural constraints to sport participation [51,52]. Scott [47] proposed a fourth category of constraint, cultural constraints, which refer to the influence of cultural norms and values on sport participation. Cultural constraints are also found to shape sport participation with demographic factors such as gender, race and ethnicity [53,54].

Recent studies have further examined the role of sport constraints in different contexts. For example, Lee et al. [55] found that technology-related constraints were a significant barrier to sport participation and argued that lack of technology skills and access to technology were important factors that limited individuals’ ability to engage in sport activities. Chen and Petrick [56] found that intrapersonal constraints were the most significant barriers to travel, followed by interpersonal and structural constraints. Similarly, Darcy et al. [57] noted that women and older adults were more likely to experience time- and family-related constraints, while men and younger adults were more likely to experience financial and work-related constraints. Although several scholars have attempted to emphasize the importance of understanding constraints in promoting sport participation, more research is needed to examine the intersection of multiple constraints and how they interact to limit sport participation [58]. In addition, it is necessary to examine how constraints vary across different domains and how they impact participation [59]. 

### 2.2. Satisfaction

Satisfaction in the context of sport refers to the contentment and positive feelings individuals derive from engaging in sport and leisure [60]. Previous research suggests that satisfaction with sport and leisure is a significant construct that is influenced by a wide range of individual, social, and contextual factors. For instance, it was found that leisure time spent with family, friends and other social networks is an important factor in increasing leisure satisfaction [61,62,63,64]. In addition, the levels of satisfaction can vary depending on demographic factors, such as age, gender, income and education level, which can all impact satisfaction [65,66]. Contextual elements, such as the accessibility of sport facilities and opportunities, as well as the specific sport activities individuals engage in, can also shape their level of satisfaction [67].

Another important finding from the literature is that satisfaction has significant implications for mental health and overall well-being. Previous studies have found that individuals experiencing high levels of leisure satisfaction show high mental wellness and low levels of stress and depression [68]. Similarly, Cho [69] explored the relationship between leisure satisfaction and well-being and highlighted that leisure satisfaction played a significant role in enhancing well-being. 

Despite these findings, however, there are research gaps and limitations that should be noted. Previous studies have used self-reported measures of satisfaction, which may be subject to biases and other limitations [65]. Additionally, there is still much to learn about the mechanisms underlying satisfaction, and more research is needed to explore the potential role of cultural factors in shaping satisfaction. Thus, further studies are of high importance to enrich this research field. In this study, we explore the sport satisfaction experiences of adult employees in Singapore. Comprehending them is vital, as greater satisfaction can foster enhanced motivation and sustained engagement in sport. Conversely, dissatisfaction, frequently stemming from constraints such as time limitations, resource deficiencies, or insufficient support, may contribute to diminished participation.

### 2.3. Nostalgia

Nostalgia can be defined as a longing or sentimental yearning for past experiences or activities [45,70]. Nostalgia has garnered attention as researchers seek to understand why people often look back on experiences with such fondness [71]. Holbrook and Schindler [72] noted that nostalgia was more likely to occur when people perceived a discontinuity between their current and past experiences, such as moving to a new location or entering a new life stage. Similarly, Cho et al. [70] found that people were more likely to experience nostalgia when they felt a sense of loss or absence in their current sport activities.

Researchers have noted that nostalgia can generate positive psychological and behavioral outcomes [45,73,74]. For instance, Kim et al. [75] found that individuals experience nostalgia for past experiences, such as going on school trips and participating in extracurricular activities. In a recent study, Cho [1] found that individuals with nostalgia showed a high level of life satisfaction and tended to engage in sport activities, suggesting that nostalgia may motivate individuals to seek out sport experiences that are meaningful and enjoyable. Additionally, inducing feelings of nostalgia has been found to increase feelings of social connectedness and reduce feelings of loneliness [76]. Likewise, Huddart and Stott (2018) noted that nostalgia could help people cope with negative life events by providing a source of comfort and familiarity. However, some researchers have cautioned that nostalgia may also have negative effects, particularly if it leads to excessive dwelling on the past or a reluctance to engage with current sport activities [77]. In sum, nostalgia is a complex and multifaceted psychological construct; there may be individual differences in how people experience nostalgia for sport activities. While many studies have found that nostalgia can have positive effects on behavioral and psychological responses, there is a need for more research to examine the underlying mechanisms of its linkage with sport experiences.

### 2.4. Sport Involvement

Sport involvement refers to the extent to which individuals engage in sport activities and the psychological significance that these activities hold for them [78,79]. In previous studies, researchers have explored various factors that contribute to sport involvement and have examined how it varies across different demographic groups. For instance, one of the earliest studies on involvement was conducted by Iso-Ahola and Mannell [80], who proposed a model of leisure involvement. They noted that people with high motivation to engage in leisure activities participate in a wide range of activities and have positive personal attributes, such as self-esteem and a sense of control over their lives [80]. Additionally, previous research has found that the availability of sport facilities and programs, as well as accessibility to transportation and information, are important factors in promoting sport participation [81,82]. Researchers have also highlighted factors that contribute to differences in sport involvement across different demographic groups [8,83] and further identified a relationship between sport involvement and diverse psychological factors, particularly its benefits for individual well-being and quality of life [84,85,86,87]. Although involvement is a complex construct that has been studied extensively in the literature, most studies have focused on the psychological and behavioral outcomes of involvement. Therefore, this study focuses on the process that leads to sport involvement to gain a broader understanding of it.

## 3. Materials and Methods

This study utilizes a qualitative research approach, which is grounded in constructivist and interpretivist paradigms. These paradigms highlight the subjective nature of reality and the significance of understanding individuals’ experiences from their own perspectives. By employing a qualitative methodology, we aimed to explore and interpret the complex, nuanced experiences and constraints of individuals in Singapore regarding their sport activities. This approach enables an in-depth examination of the participants’ personal narratives, yielding rich, detailed insights that may not be captured through quantitative methods.

### 3.1. Data Collection and Participants

To recruit research participants, this study employed a combination of snowball and purposeful sampling. Initially, we utilized social media platforms and sport community groups to identify individuals who expressed a high intention to participate in sport activities but were experiencing constraints. Additionally, participants were required to work more than 45 h per week for inclusion in this study. Before collecting data, this study obtained Institutional Review Board (IRB) approval from a university in Singapore. Purposeful sampling allowed us to select participants who met specific criteria relevant to our study, ensuring a focused and relevant sample. Participants were chosen based on their self-reported decrease in sport participation due to various constraints. Once initial participants were identified, snowball sampling was employed to expand the sample. For example, Darren (male, 44) introduced Fiona (female, 44) and Tom (male, 49) introduced Perry (male, 36) and Jane (female, 36). This method helped us reach additional participants who fit our criteria through referrals from the initial participants. 

We conducted semi-structured interviews, crafting questions along the topics of constraints and sport experiences to gather their past and current experiences in sport. The interview questions were structured to explore the main research question while allowing for unanticipated themes to emerge [88]. The interview protocol was developed through a collaborative process to ensure its theoretical and practical robustness. The initial draft was created by the first author based on a thorough review of the relevant literature and research objectives. This draft was reviewed by a panel of four experts in the fields of sport psychology, sport management and leisure. They provided feedback on relevance and clarity of the questions. It consisted of semi-structured questions designed to explore various facets of the participants’ experiences, including introductory (e.g., what is the most frequent or enjoyable leisure activity that you participated in?); constraint-related (e.g., what factors have affected your ability to participate in sport activities recently?); attitude and negotiation (e.g., how has your participation in sport activities changed over time?); nostalgia (do you feel nostalgic about any past sport experiences? If so, how does this nostalgia affect your current participation?); and future involvement (e.g., what are your future plans or goals related to sport activities?) themes. The interview guide was customized to elicit in-depth responses on these key areas, while also allowing flexibility to investigate emerging topics as necessary. 

All interviews were recorded using an audio recording device to facilitate efficient transcription. Each interview lasted between 20 to 40 min and was conducted at the convenience of the interviewee, either at their residence or a nearby café. The average duration of the interviews was 32.67 min, with a standard deviation of 6.25 min. Although one interview lasted only 20 min, we ensured that sufficient information was collected through a combination of well-structured questions and a flexible interview approach that allowed participants to elaborate on their experiences. Each interview was designed to cover all the essential topics related to constraints and sport experiences while allowing for deeper exploration based on participants’ responses.

The final selection of participants was based on achieving data saturation. Specifically, we determined that data collection and analysis had reached saturation through an iterative process. We continuously compared new data to existing themes using the constant comparison method, until no additional themes emerged [89]. In other words, data saturation was considered to have been achieved when further interviews did not uncover new information or themes, existing themes were consistently supported by new data, and peer debriefing sessions confirmed that saturation had been reached. In total, 10 participants residing in Singapore were interviewed. These participants consisted of seven males and two females, aged between 33 to 49 years (Table 1). Among them, nine were married with children and one was single. The majority of participants held a college degree and had an average working hour per week of 53.3 h. 

After the interview, the recordings were transcribed, and participants were provided with the details of the study, including the purpose, confidentiality, time frame, waiver and contact information. We also explained the study to the participants to ensure a better understanding of the research. Contact information, including the participant’s contact number and email address, was collected for future communication if needed.

### 3.2. Data Analysis

To provide an archive of a more specific targeted group for future research with similar topics [90], the interviews and analysis of the transcripts from the collected data were conducted simultaneously using the constant comparison method [91]. This methodological approach was chosen because it systematically compares data elements, helping to develop and refine concepts and categories through an iterative process. This aligns well with our constructivist and interpretivist research paradigms, which emphasize understanding participants’ subjective experiences and constructing theory grounded in their perspectives. During the analysis, the transcripts were examined in detail to gain a deeper understanding of the participants’ responses. The data analysis involved three coders to ensure a rigorous thematic analysis. Coding was placed along the margins of the document in alignment with the transcript text. This process was repeated for the other transcripts, with the audio files being played several times to ensure accuracy. The location and time of the interview were labelled in each transcript. The main themes drawn from the responses were compiled and compared to the original transcripts of the participants to ensure validity. Further, to ensure trustworthiness, we conducted member checking, wherein preliminary findings were shared with participants for validation. We returned the findings to the participants to confirm the accuracy of the data and interpretations. Additionally, peer debriefing sessions were conducted to validate the data analysis. Specifically, we shared our findings with five professors in the field of sport psychology to receive their feedback on the interpretations. Data triangulation was used by selecting participants from diverse sectors and age groups to provide a broader range of experiences. Additionally, theory triangulation was applied by integrating perspectives from emotion-related and leisure constraint theories to offer a comprehensive understanding of the role of nostalgia in sport participation. Finally, the themes were categorized with the research topic to evaluate the data. In particular, the main topic of this study is related to constraints. Therefore, this study adopted a deductive approach, utilizing Crawford and Godbey’s [92] constraints model to classify the qualitative data. This model categorizes leisure constraints into intrapersonal, interpersonal and structural constructs. We used this framework to organize and interpret our findings, providing a structured lens through which to analyze the constraints experienced by participants.

## 4. Results

The aim of this study was to investigate constraints and sport experiences among individuals in Singapore. We identified four main themes from the interviews: *factors causing the reduction of sport opportunities, changes in sport activities and satisfaction, nostalgic feelings associated with sport activities and the role of nostalgia in enhancing sport involvement*. Each theme highlights different aspects of participants’ sport experiences and constraints. 

### 4.1. Factors Causing the Reduction of Sport Opportunities

Participants reported various constraints that contributed to a reduction in their sport participation. A common factor was physical limitations due to injury or aging. For example, Peter (33, male) described how an ACL injury reduced his soccer participation from once a week to once a month, due to limitations in his ability to play and the need to manage his injury. Additionally, another participant, Jet (42, male), had started playing badminton when he was six years old and had enjoyed the activity with his family and relatives in his younger days. However, as Jet grew older, he found that his recovery period after badminton games had significantly slowed down, preventing him from maintaining the high intensity of the games he used to enjoy. Even though Jet still played badminton once a week, he was not able to enjoy it as much as before due to his physical constraints and the natural aging process. The psychological fear of getting injured and the long recovery period associated with aging were the barriers that Jet did not want to go through. That is, aging affects his ability to play badminton at high intensity, leading to reduced enjoyment and participation:

*Around 3–4 years ago, I suffered an ACL injury, and it doesn’t mean that I can’t play. I still can, but there were limitations to it. So that is the reason why I have to reduce the playing time*.

(Peter)

*As you grow older, injuries and recovery are much slower than before, so you don’t expect to play at very high intensity when you haven’t been doing it frequently. One of my biggest worries is also injuries and age*.

(Jet)

Environmental constraints such as family and work commitments also played a significant role. Darren (44, male) emphasized the difficulty of balancing family responsibilities with sport participation, particularly when considering the risk of injury:

*We still have a family to take care of. I can’t afford to play a football game, and then tomorrow, tell my boss that I will be on medical leave for a week because of a sprained ankle, even though it is very hard to justify that*.

(Darren)

Apart from age, other factors, such as the availability of facilities, family commitments and work commitments, have also limited the sport participation of participants like Darren and Jet. Previous studies on constraints have also identified similar factors, including a lack of time, money, and information [40]. While this study’s findings showed similarities in terms of time constraints, other constraints, such as insufficient information and economic affordability from Scott et al.’s [40] study, were not applicable in the context of Singapore.

Another participant, Perry (male, 36), who had been actively trekking for the past 7–8 years, saw a significant decrease in his engagement in this sport activity due to social factors, such as the personalities of other participants and interactions between them during his sport activity. Perry found it challenging to deal with multiple inputs from different people, and friction within the group of friends that was inevitable due to the nature of the activity itself.

*Different people have different personalities, so when you explore and you go trekking with a different group of people, different inputs will come in, so that’s the part that so-called the organizer has to handle*.

(Perry)

In addition, another psychological factor that led to a reduction in sport participation was pressure. Tom (49, male), an avid golf player who has been playing golf for around 25 years, mentioned that stress sometimes made him reconsider his participation in the sport activity as he could not enjoy the game if he was under too much pressure. Overall, constraints such as physical limitations, psychological constraints, environmental factors and social dynamics were key reasons for reduced sport participation.

### 4.2. Changes in Sport Activities and Satisfaction

Participants also reported changes in their sport activities and associated levels of satisfaction. For instance, Darren (44, male) transitioned from playing football to watching soccer due to physical constraints, resulting in dissatisfaction with his current level of engagement:

*Although my passion for football remains, I have shifted to watching soccer due to physical constraints. The alternative of watching the sport isn’t as fulfilling as participating*.

(Darren)

Jet (42, male) similarly experienced dissatisfaction due to limitations on court availability, which prevented him from playing badminton as often as he would like. Despite these changes, some participants found new forms of enjoyment. Jane (36, female) and Mark (40, male) expressed positive outcomes from their sport activities, such as social bonding and stress relief:

*I enjoy the competitive element in badminton and the social interactions with friends during leisure time*.

(Jet)

*Bowling provides a break from work and family, allowing me to enjoy the sport without worrying about standards*.

(Mark)

For these participants, the outcomes of their sport activities were the main motivators for maintaining their lives. During follow-up interviews, positive feelings were expressed, with some participants recalling specific details of their sport experiences vividly from memory.

### 4.3. Nostalgic Feelings Associated with Sport Activities

The theme of nostalgia emerged strongly, highlighting how past sport experiences evoke significant emotional responses among participants. This theme not only reflects their personal experiences but also how their current circumstances, including family dynamics, influence their nostalgic feelings. Several participants expressed vivid nostalgic memories related to their past sport activities. Perry (36, male), an avid trekker, shared detailed recollections of a memorable trek, describing the landscape and environmental conditions that evoked a deep sense of nostalgia:

*We went during autumn, so we shouldn’t see any snow, but when we reached the base camp which is around 4900 m above sea level, we managed to see snow and the whole scenery really resembles the north pole, basically those houses painted blue on the rooftop. You see the whole place in white, it was nice, really nice*.

(Perry)

This sense of nostalgia was further amplified when Perry reflected on past experiences and how they shaped his current motivations for trekking. His nostalgia was not only about the physical experiences but also the emotional connections formed during these treks. 

Darren (44, male) provided an additional dimension to the theme of nostalgia by relating his past sport experiences to his present life through his children. He mentioned watching his son play soccer, which evoked nostalgic feelings as he recalled his own days of playing soccer:

*Seeing my son play soccer reminds me of my own days on the field. It is a mixed feeling of joy and longing for those times when I was actively playing*.

(Darren)

This reflection illustrates how observing his son’s participation in soccer rekindled Darren’s own memories of the sport, blending his past with his current experiences.

Similarly, Tom (49, male) experienced nostalgia as he used to play golf once a week when he was younger, but the time he spent on golf gradually decreased as he aged due to other commitments. Although his time for golf diminished, he found joy in seeing his children partake in sport, which provided him with a sense of connection to his own past:

*Even though I don’t play golf as often, watching my children take part in sport brings back memories of when I was actively involved. It’s a way of reliving those experiences through them*.

(Tom)

Another form of nostalgia expressed by participants was related to their social interactions during their sport activities. Jane (36, female) expressed her longing for past badminton training sessions, where she and her classmates would gather on weekends and enjoy each other’s company.

*As I said, I spent a lot of time training, so I missed the time I spent with my own classmates. When my classmate gathers together on the weekend, I am unable to because I have the training to attend to*.

(Jane)

Cho et al. [93] noted that nostalgia can develop from objects and interpersonal relationships, while Korte [94] stated that socialization can increase an individual’s sense of identity, leading to the development of nostalgia for group identity. Tajfel’s [95] view on social identity suggests that an individual’s emotional attachment to a specific group establishes a sense of belonging, and Jane’s expression during the interview evoked those feelings. Specifically, nostalgia for group identity surfaced during Jane’s representation of her school team’s badminton tournament, highlighting her motivation and continued participation in badminton. This study also found that a participant’s sport experience is associated with nostalgia for personal identity. For example, Perry took pride in being an avid trekker and had nostalgic feelings toward his personal identity. He also felt nostalgia for the time, effort and resources he had invested in the activity.

### 4.4. The Role of Nostalgia in Enhancing Sport Involvement 

This section specifically explores how nostalgic feelings impact participants’ current involvement in sport activities. While previous sections discussed constraints and general changes in sport participation, this section focuses on how nostalgia not only triggers past memories, but also motivates continued or renewed engagement in sport.

Nostalgia played a significant role in motivating participants to remain involved in or return to sport. For example, Perry (36, male) experienced a strong connection between his nostalgic memories of trekking and his motivation to continue the activity. Perry missed those sport experiences and the valuable feedback he received, which he regarded as lifelong tips. Although he cannot turn back time, the feedback he gained had a lasting impact on his current trekking skills. This indicates how past sport involvement can affect current sport involvement. In addition, Perry experienced nostalgia due to the unique experiences of each trek, which helped motivate him to continue his passion for trekking. It suggests that nostalgia can enhance current sport involvement by providing positive memories and motivation to continue participating in sport activities.

*I would say I have the feelings because every trek is different. I think that is the key part of trekking. So, when I look back at this mountain or that mountain, I feel nostalgic. It is related to how that particular trip is heavy or how eventful*.

(Perry)

However, Perry also noted the need for lifestyle changes to fully enjoy his sport activities, suggesting that nostalgia alone may not be sufficient to sustain involvement.

Nostalgia also influenced participants’ choices of sport activities. Darren (44, male), although physically constrained from playing soccer, expressed a renewed connection to the sport through watching his son play. This experience underscored how nostalgia for his own soccer days fueled his ongoing interest. Similarly, Tom (49, male) found that nostalgic feelings associated with his past golf activities helped him stay engaged with the sport, even if his participation had decreased:

*Watching my son play soccer reminds me of my own playing days. It’s a joy to see him and relive those memories through him*.

(Darren)

*I don’t play golf as often, but the memories and seeing my children in sport remind me of my love for it. It’s a way of holding onto those past experiences*.

(Tom)

Nostalgia sometimes helped participants overcome the constraints that limited their sport activities. For instance, Jane (36, female) used her nostalgic memories of past badminton training sessions to sustain her interest in the sport, despite current barriers:

*I miss the social interactions and training sessions. These memories keep my interest in badminton alive, even though I can’t participate as much*.

(Jane)

Overall, the findings suggest that nostalgic feelings have a significant impact on one’s sport involvement in the present, influenced by specific sport experiences and past involvement levels. Some participants expressed their willingness to change their current lifestyles to enhance their sport experiences. The constraints that trigger nostalgia and motivate participants to make changes can ultimately lead to an increase in current sport activity participation and a more positive mindset toward participating in sport activities. Therefore, constraints may trigger nostalgia, encourage motivation, diminish the perception of constraints and facilitate negotiation efforts, ultimately leading to positive actions toward sport participation.

## 5. Discussion

This study aimed to identify the types of constraints and their relationship with satisfaction, benefits of sport, nostalgia and sport involvement among Singaporean adults, as there are limited studies on constraints among this population. In this study, we identified four main themes related to sport experiences and constraints among individuals in Singapore. These themes provide insight into the various factors affecting sport participation and the role of nostalgia. The first theme, *factors causing the reduction of sport opportunities*, indicates that participants experienced certain constraints, which resulted in negative emotions and dissatisfaction with their limited engagement in sport activities. The second theme, *changes in sport activities and satisfaction*, highlights the fact that participants highlighted multiple advantages that they could gain from sport. The third theme, *nostalgic feelings associated with sport activities*, refers to nostalgia related to these sport activities. Finally, the last theme, the role of nostalgia in enhancing sport involvement, focus on how nostalgic feelings influence participants’ current sport involvement and how they can change individuals’ current lifestyle to continue their sport activities. We discuss these themes in detail, exploring how each contributes to our understanding of sport participation and the influence of nostalgia.

The constraints identified in this study were classified into four main groups: physical, environmental, psychological and social factors. To contextualize these constraints within existing theoretical frameworks, we used Crawford and Godbey’s [92] constraints model, which classifies leisure constraints into three constructs: intrapersonal, interpersonal and structural. Physical and psychological constraints belong to the intrapersonal construct. These constraints include personal health issues and psychological barriers, which directly impact an individual’s ability to engage in sport. Social factors, such as family commitments and social interactions, align with the interpersonal construct. These constraints relate to how interactions with others affect one’s participation in sport. Environmental factors, including facility availability and work commitments, correspond with the structural construct. Structural constraints involve external barriers that could be addressed through policy changes, such as adjustments to working hours or improved access to sport facilities. Our findings indicate that addressing structural constraints, particularly those related to working hours, could help Singaporean adults engage in more sport activities, leading to improved satisfaction, reduced stress and better health outcomes. For instance, participants highlighted work commitments and family responsibilities as significant barriers to sport participation. Singaporeans work more hours per week compared to individuals in some other countries, resulting in less time for sport and increased stress. Hence, revising working hour policies or encouraging companies to implement flexible working arrangements could enhance quality of life and reduce health issues among Singaporean workers.

However, this may present a compromise for workers who have to choose between income and leisure time. Participants often expressed a sense of being torn between earning a livelihood and having time for leisure activities, including sport. This tension is not unique to Singapore but is also seen in other countries, such as Australia, where work-time reduction is a topic of debate [96]. According to Gerold and Nocker [96], workers’ working-time preferences are shaped by social norms (i.e., full-time working norm) and personal priorities, such as the need for financial security versus the desire for leisure time. Furthermore, participants in this study expressed dissatisfaction with their current situation, where they could not maintain their sport participation as they used to. This dissatisfaction is consistent with Hughes and Bozionelos’ [97] findings that work–life imbalance is a significant source of dissatisfaction among workers, leading to labor turnover and other work withdrawal behaviors.

Work–life balance refers to the balance of work, whether done on the job or at home, and leisure time for working individuals to enjoy life to its fullest [41]. In contrast, individuals who feel in control of their work are more likely to perceive a balanced life. Mitigating structural impediments, particularly those pertaining to working time arrangements, could substantially improve the quality of life for Singaporean adults. Introducing policies that foster a better equilibrium between professional and personal domains, such as flexible or reduced work schedules, may stimulate increased engagement in sport activities. This shift could yield enhanced job satisfaction, diminished stress and improved health outcomes. Incentivizing organizations to implement these policies and disseminate the corresponding findings may represent a strategic approach to elevating the overall well-being of the workforce.

Another significant finding of this study is the relationship between constraints and nostalgia among Singaporean adults. Individuals encountering physical and psychological barriers to sport participation may experience an increased sense of nostalgia, as they look back on past sport activities in the context of their present limitations. This feeling of nostalgia can act as a motivational factor, alleviating some of the constraints and potentially fostering renewed interest in sport. By exploring this dynamic, we offer a deeper insight into how nostalgic emotions can affect sport engagement, despite the constraints outlined in our study.

Participants’ nostalgic feelings toward their sport activities were associated with their past experiences, social interactions and personal identity. The conceptual model of nostalgia by Cho et al. [93] can be incorporated into the findings of this study. The model was designed to explain the feelings of nostalgia through a scope of memories, and Cho et al. [1,70] further provided five different aspects of nostalgia (i.e., experience, environment, socialization, personal identity and group identity) and discussed how those nostalgic feelings affect one’s sport activity. Individuals’ nostalgia regarding past sport participation is not simply a yearning for past activities, but rather encompasses a multifaceted interplay of experiences and emotions that influence their current behaviors.

Participants frequently reflected on their younger years, during which they engaged in sport alongside friends, enjoyed communal spaces and experienced a profound sense of belonging. For example, one participant reminisced about playing soccer in the past, emphasizing how these memories evoke nostalgic feelings. This nostalgia motivates them to seek analogous experiences in their present-day lives, though these may be modified by their current circumstances. In addition, the role of nostalgia in sport participation may vary across cultural contexts. For example, the nostalgia associated with outdoor ice rinks in Canada appears to be a significant attraction for sport tourists, evoking a sense of place identity and an idealized past [98]. In the American context, research suggests that sport halls of fame and museums also serve as sites of collective and private nostalgia, allowing visitors to engage with cherished memories and a romanticized vision of sporting history [99]. These nostalgic memories can inspire continued engagement in physical activities and sport, as individuals seek to reconnect with their personal or cultural past.

Moreover, the level of an individual’s participation in sport activities in the past can be used to predict their current frequency of sport activity. Specifically, sport activities can be classified based on the level of commitment by applying the six different features of serious leisure pursuits from the past [100]. These include perseverance, significant personal efforts, unique ethos, strong identification with the activity, durable individual benefits and involvement in a career. Stebbins’ [100] model enables a better understanding of the relationship between past leisure activities and their impact on current involvement, contrasting casual and serious leisure. As serious leisure is considered significantly more rewarding and fulfilling compared to casual leisure, participants with such a level of commitment are more likely to overcome the adversity posed by the various costs of that leisure, suggesting a higher level of participation in leisure in the future.

This study has contributed to the understanding of constraints and sport experience among individuals in Singapore and the level of commitment to their sport activities in the past, predicting their sport activities in the present or future. Moreover, understanding the role of nostalgia in sport participation can provide practical insights for policymakers and sport organizations. For example, organizations could incorporate nostalgia into wellness programs by creating opportunities for employees to engage in activities that evoke positive past experiences. Additionally, developing community programs that revive traditional or culturally meaningful sport activities could leverage nostalgic feelings to enhance participation. Similarly, in the Singaporean context, programs that incorporate traditional games or sport familiar to older adults could potentially foster greater engagement among this demographic. Finally, we recommend that policymakers develop targeted strategies to overcome barriers that hinder employee engagement in sport and physical activities. Suggested interventions might involve introducing flexible work hours or establishing on-site fitness centers, which could alleviate the adverse effects of high-pressure work settings and contribute to improved employees’ well-being.

### Limitations and Future Research

This study has some limitations that should be considered. The qualitative approach used in this study provides insights into the subjective experiences of Singaporean workers. However, it also faces certain methodological limitations. Relying on self-reported data may introduce biases, as participants may not fully or accurately recollect their experiences. Moreover, qualitative findings from a single cultural context can be challenging to generalize. Additionally, the heterogeneity in participant age, occupation and other characteristics may have contributed to variability in the study’s findings, while this diversity could limit the generalizability of the results to broader populations. Future research should explore a more homogeneous sample or utilize additional methods to account for these demographic factors. In addition, increasing the sample size and conducting diversified studies across countries can facilitate higher generalizability for this research. Second, the findings of this study suggest that nostalgia can be a powerful motivator for sport participation, but its manifestations and impacts can differ significantly across cultures. Thus, future research should explore these cultural intricacies to develop a more comprehensive understanding of how nostalgia influences sport participation globally. In addition, by being more specific and looking into each constraint meticulously, researchers might be able to locate new findings, and it will benefit the understanding for future studies on sport-specific nostalgia.

## 6. Conclusions

According to Sport Singapore [26], 51% of participants do not participate in sport activities due to their long working hours, indicating that working adults in Singapore seldom engage in sport activities because of their long working hours. However, it should be noted that life satisfaction, physical and mental health have positive relationships with outdoor, physical and social activities. If constraints continue to dominate an individual’s life, it will lead to negative health and wellness consequences, and the number of inactive individuals in sport activities would gradually increase. Since nostalgia is generated by sport activities that cannot be enjoyed currently, it motivates and increases individuals’ willingness to participate in their preferred sport activities. That is, the findings highlight the significant role of nostalgia as a motivational force for adult workers facing barriers to sport participation. By reconnecting individuals with positive memories of their past sport involvement, nostalgia acts as an emotional catalyst, encouraging them to renew their interest and commitment to physical activity. This suggests a valuable application for sport promotion strategies, where nostalgia is intentionally employed to motivate adults to overcome present challenges and re-engage in active lifestyles. Future research could explore the nostalgic feelings within each of the four constraints discussed in this study, including physical, environmental, social, and psychological constraints. The findings of this study provide a foundation that shows the current constraints faced by Singaporean working adults and how these constraints affect their nostalgia and involvement. By extending the research on each constraint, researchers may be able to identify new findings or emerging themes within each factor itself, which would contribute to a better understanding for future studies on sport-specific nostalgia. Additionally, this study encourages the government, private and public organizations to take further action to develop policies that facilitate participation in sport activities for workers in the long term.

## Figures and Tables

**Table 1:** Characteristics of participant.

Name	Age	Gender	Marital Status	Presence of Children	Occupation	Working Hours per Week	Education
Perry	36	Male	Married	Yes	Financial Service Manager	50	Bachelor’s degree
Jane	36	Female	Married	Yes	Account Executive	45	Diploma
Tom	49	Male	Married	Yes	Remisier	40	Bachelor’s degree
Darren	44	Male	Married	Yes	Deputy Chief Executive Officer (CEO)	50	Bachelor’s degree
Fiona	44	Female	Married	Yes	Manager	50	Bachelor’s degree
Mark	40	Male	Married	Yes	Chief Operating Officer	50	Master’s degree
Jet	42	Male	Married	Yes	Sport Manager	40	Bachelor’s degree
Ali	44	Male	Married	Yes	Taxi Driver	45	Secondary School
Peter	33	Male	Single	No	Claims Executive	40	Bachelor’s degree
George	63	Male	Married	Yes	Taxi Driver	70	Secondary School

## Data Availability

The data presented in this study are available on request from the first author. The data are not publicly available due to privacy.
